# Psychological and Antibacterial Effects of Footbath Using the *Lindera umbellata* Essential Oil

**DOI:** 10.3390/molecules26175128

**Published:** 2021-08-24

**Authors:** Maiko Kitajima, Marika Miura, Naoki Nanashima, Toshiko Tomisawa, Shizuka Takamagi, Miyuki Fujioka, Naoya In, Tomohiro Osanai

**Affiliations:** 1 Department of Nursing Sciences, Hirosaki University Graduate School of Health Sciences, 66-1 Hon-cho, Hirosaki 036-8564, Aomori, Japan; mm4679@gmail.com (M.M.); tmtott@hirosaki-u.ac.jp (T.T.); takamagi@hirosaki-u.ac.jp (S.T.); in1105@hirosaki-u.ac.jp (N.I.); osanait@hirosaki-u.ac.jp (T.O.); 2 Department of Bioscience and Laboratory Medicine, Hirosaki University Graduate School of Health Sciences, 66-1 Hon-cho, Hirosaki 036-8564, Aomori, Japan; nnaoki@hirosaki-u.ac.jp (N.N.); mfujioka@hirosaki-u.ac.jp (M.F.)

**Keywords:** aromatherapy, *Lindera umbellata*, footbath, autonomic nervous system activity

## Abstract

*Lindera umbellata* (*Lu*) essential oil primarily contains linalool and has relaxation properties. We investigated the psychological and antibacterial effects of footbath with *Lu* essential oil. The participants included 20 women without medical history and received two intervention plans: footbath without any essential oil and footbath using *Lu* essential oil. Next, questionnaires regarding impressions and mood states were provided for them to answer. In addition, their autonomic nervous system activity was measured, and the aerobic viable of count on the feet was determined. The high-frequency value reflecting the parasympathetic nervous system activity significantly increased after footbath using *Lu* essential oil. In the questionnaire about the mood states, the subscale scores of tension–anxiety, depression, fatigue, and confusion after intervention were lower than those before intervention regardless of the use of the essential oil. Conversely, the anger–hostility score decreased only in the group using *Lu* essential oil. Furthermore, the decrease in aerobic viable count after intervention was not significantly different between the two groups. Footbath using *Lu* essential oil increased the parasympathetic nervous system activity and relieved anger. Taken together, we suggest that footbath using *Lu* essential oil has a relaxation effect.

## 1. Introduction

Essential oils are natural products from a variety of plants and are generally used for fragrance and massage purposes. In several studies, some researchers have revealed the physiological functions, mechanisms, and physiological effect (i.e., sedative and anxiolytic) of essential oils [1,2,3].

*Lindera umbellata* (*Lu*) is a deciduous shrub growing mainly in northeast Japan and the essential oil is made from its leaves and/or branches by steam distillation. It consists mostly of linalool, which has been suggested in some studies to have anxiolytic and antidepressant effects [4]. Maeda et al. reported that linalool in *Lu* can be regarded as a natural resource for use in anti-inflammatory and anticancer therapeutic products [5,6]. However, the physiological and antibacterial effects of the *Lu* essential oil in humans remain to be clarified.

In nursing care, some nurses provide footbaths using various essential oils as one of the alternative complementary therapies. Gnatta et al. proposed that aromatherapy could be a nursing intervention to improve the comfort of patients as well as their family or community [7]. Footbaths are one of the significant nursing procedures to improve patient sleep, relieve fatigue, and clean their feet for the prevention of infections [8,9]. Saeki reported that footbath with lavender essential oil affected the activity of the autonomic nervous system [10]. In patients with stroke, it is reported that back massage with a blended essential oil and footbath reduced their stress and changed their mood states [11]. Therefore, we expected that footbath with the *Lu* essential oil may be useful for relaxation. In this study, we investigated the psychological and antibacterial effects of footbath with and without the *Lu* essential oil.

## 2. Results

### 2.1. Changes of the Autonomic Nervous System Activity

**Figure 1:**
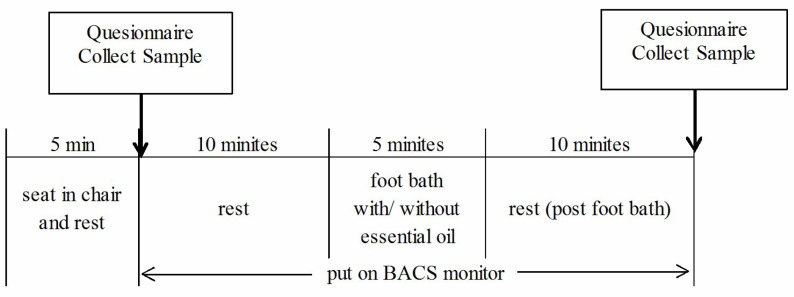
Research protocol.

Figure 1 shows the protocol of this study (the detail of protocol is seen in Section 4.4. *Protocol* in **Materials and Methods**). In brief, at first, the participants sat on a comfortable chair and rested for 5 min to prevent the influence of any previous action. Then, they filled out the questionnaires, stamped their big toe on the agar medium, and put their right index finger on the BACS monitor for 10 min. Subsequently, they placed their feet in the footbath equipment and randomly received footbath for 5-min with the *Lu* essential oil (i.e., L group) or without the *Lu* essential oil (i.e., C group). After the intervention, the participants sat for 10 min. Finally, they completed the questionnaires and stamped their big toe on the agar medium again.

**Figure 2:**
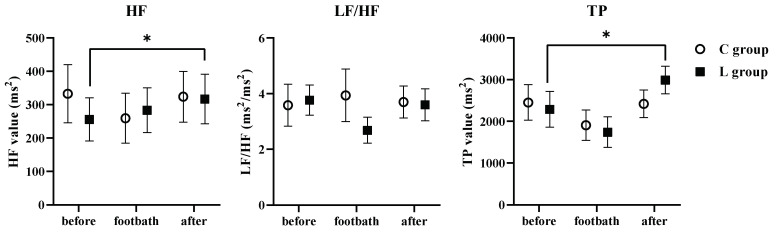
Changes in the HF, LF/HF, and TP values by footbath with or without *Lu* essential oil (n = 20). Before: before taking a footbath (Before the intervention), Footbath: during taking a footbath, After: after taking a footbath (after the intervention). HF: High-frequency value, LF/HF: Low-frequency/high-frequency value ratio, TP: Total power. The HF power spectrum is 0.15–0.4 Hz and reflects the parasympathetic nervous system activity. * *p* < 0.05 between before and after using repeated measures ANOVA and Bonferroni test.

Figure 2 shows the results of the BACS monitor. The BACS monitor was put on the participants’ right index finger, recorded the heart rate through pulse wave detection, and automatically calculated the high-frequency (HF), low-frequency (LF)/HF ratio, and total power (TP). The LF value reflects the activities of both the sympathetic and parasympathetic nervous systems. On the other hand, the HF power spectrum only reflects the parasympathetic nervous system activity. The LF/HF value ratio indicates the parasympathetic dominance, and the TP is the power of the adapting autonomic nervous system. In the L group, the HF and TP values after intervention were significantly higher than those before taking a footbath (both values, *p* < 0.05), whereas this phenomenon was not observed in the C group. Taken together, it shows that the function of the parasympathetic nerve predominates by using *Lu* essential oil. There was no significant difference between the LF/HF values before and after the intervention in both groups (Figure 2).

### 2.2. Effect of the Psychological Index

The Profile of Mood States (POMS) subscale scores of the patients are presented in Table 1. The POMS assesses transient mood states and measures six mood or affective states: ‘tension–anxiety’, ‘depression–dejection’, ‘anger–hostility’, ‘vigor’, ‘fatigue’, and ‘confusion’. In addition, the total mood disturbance (TMD) score indicates the negative feeling of participants from the POMS score.

The ‘tension–anxiety’, ‘depression–dejection’, ‘fatigue’, ‘confusion’, and ‘TMD’ scores significantly decreased after the intervention in both the C and L groups (*p* < 0.05). In addition, the ‘anger–hostility’ score was notably reduced only in the L group (*p* < 0.05). This result suggests that *Lu* essential oil produces additional effects.

After the intervention, the degrees of comfort, relaxation, and fatigue between the groups were not significantly different (1.10 ± 0.31, 1.35 ± 0.49, and 1.35 ± 0.59 in the C group, 1.05 ± 0.22, 1.20 ± 0.41, and 1.10 ± 0.31 in the L group, respectively). The participants answered regarding their impression of footbath and aromatherapy as well as their feelings toward the footbath with or without the essential oil after each intervention. In the ‘impression of footbath’, 11 participants answered ‘to be able to relax’, four noted ‘clean’, and three responded ‘promote blood circulation’ and ‘promote sleep’. With regard to the impression about the essential oil, 14, six, and five participants answered ‘relaxation’, ‘nice fragrance’, and ‘become calm’, respectively. Moreover, pertaining to their feelings after each intervention, some participants answered ‘comfortable’ and ‘warm’. In the L group, they answered ‘nice fragrance’, ‘the fragrance of the *Lu* essential oil was comfortable and not too strong’, and ‘the mind was calmed by the fragrance’.

To the question ‘Do you use footbath with essential oil in your nursing care?’, 15 nursing students answered ‘yes’. Based on the responses, it has relaxation and refreshing effects, and is thought to be beneficial to the patients who are unable to take a bath. However, it is challenging for subjects to correspond individually following each favorite for many essential oils and to prepare a space to use essential oils.

### 2.3. Aerobic Viable Count

**Figure 3:**
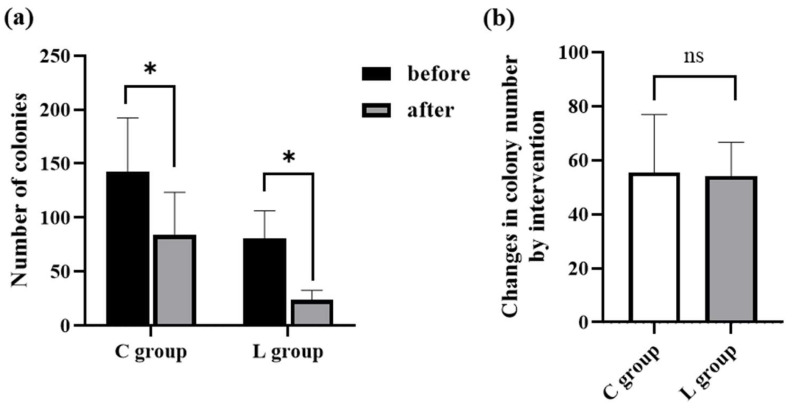
Changes in the number of aerobic colonies by footbath with or without *Lu* essential oil (n = 20). (**a**) Number of aerobic colonies before and after the intervention in the C group and L group. Before: Before taking a footbath, after: after taking a footbath. * *p* < 0.05. (**b**) Changes in colony number of the C and L groups, which were calculated by subtracting the colony number after intervention from that before the intervention. ns means *p* > 0.05.

As shown in Figure 3a, the aerobic viable count significantly decreased after the intervention in both groups (*p* < 0.05). Next, we investigated whether there was a difference in anti-bacterial effect between the C group and L group. Since the number of aerobic colonies before the intervention differed between the C group and L group, we compared the changes in colony number of each group, which were calculated by subtracting the colony number after intervention from that before intervention. As a result, there was no significance between the C group and L group (Figure 3b). Taken together, these results suggest that although the footbath itself clearly decreased the aerobic viable count, the addition of *Lu* essential oil failed to further decrease the aerobic viable count.

## 3. Discussion

Essential oils have various effects on the human body through inhalation and percutaneous application. The inhalation of essential oils can send signals to the olfactory system and stimulate the brain to exert neurotransmitters, thereby further regulating the mood [12]. Footbaths with an essential oil such as in this study are considered to have such effects through both inhalation and percutaneous application. To our knowledge, this is the first study to evaluate the effects of using the *Lu* essential oil on humans.

We found that footbath with the *Lu* essential oil significantly elevated the HF value, namely a parasympathetic nerve predominant state. In addition, since the TP value also increased after the intervention, it is thought that the autonomic nervous activity was stabilized by intervention. The scores of the POMS questionnaire and subjective feelings showed that the footbath with or without the essential oil significantly decreased a negative mood state. It has been reported that footbath itself can change a negative mood state such as anxiety [13,14]. Therefore, the result that some POMS scores such as tension–anxiety and depression–dejection were significantly changed in the C group as well as L group may be explained by the effect of the footbath itself. Of note, the footbath with the *Lu* essential oil additionally decreased feelings of anger (e.g., furious). The feeling of anger is known to be a risk factor for stroke and coronary heart disease [15,16]. Therefore, a decrease in the feeling of anger through interventions such as footbaths using essential oils may be beneficial for patients with those diseases. Overall, we concluded that the use of the *Lu* essential oil may provide psychological and emotional benefits.

We recently reported the chemical composition of *Lu* essential oil [17]. Linalool is the main component of the *Lu* essential oil used in this study (42.8%), followed by 1.8-cineole and D-limonene (13.7% and 7.42%, respectively; Figure S1). Linalool is known to have analgesic, anxiolytic, and antibacterial properties, and the inhalation of an essential oil with linalool has been demonstrated to be associated with relaxation [4,18,19]. Similar to our research, Saeki reported that footbaths with lavender oil, which mainly contains linalool [20], has a relaxation effect [10]. Therefore, it is possible that the relaxation effect of *Lu* essential oil is due to the effect of linalool, and there is a possibility that other essential oils containing linalool may elicit similar effects observed in this study.

Prior to the intervention, no significant difference in the aerobic viable count between the two groups was noted; however, it was significantly reduced after footbath. Most of the essential oils extracted from plants have been reported to have a potent antimicrobial activity in vitro and be effective against various bacteria [21,22,23]. Some essential oils containing mainly 1,8-cineole have been reported to inhibit the growth of some pathogenic bacteria [24,25,26]. Although the *Lu* essential oil also contains 1.8-cineole [17], there was no significant difference in the antibacterial effect between the C group and the L group. The concentration of the *Lu* essential oil may be able to explain this result. In brief, the concentration used in this study was <1% of the total because a *Lu* concentration of >1% had a too strong smell and was hyper-stimulated to the skin for the participants. Thus, to clarify the antibacterial effect of *Lu* essential oil, we need to investigate whether footbaths using a higher concentration of the *Lu* essential oil show an antibacterial effect in a future study.

Footbaths with essential oils for patients can be expected to clean their feet and give relaxation by tailoring them to their preference. Aromatherapy is sometimes a difficult procedure because of the unknown effects of some essential oils. Nonetheless, a few studies have reported this intervention in postoperative and palliative care [27,28,29,30]. Therefore, we believe that sufficient evidence on the effects of essential oils contributes to the effective use of aromatherapy.

## 4. Materials and Methods

### 4.1. Participants

The participants in our study included 20 female university students aged 21.45 ± 0.69 years without medical history and foot injury. They underwent a skin allergy test for the *Lu* essential oil before the intervention to prevent some of the most common adverse events, for example, skin irritation. No adverse events occurred in all participants. Furthermore, they were satisfied with the flagrance of the *Lu* essential oil. This is significant because the participants’ discomfort with the fragrance may affect the psychological index results [31].

### 4.2. Setting

The test room was quiet, and the temperature was adjusted to 24–26 °C. All interventions in this study were performed from August 2018 to September 2018. We checked the vital signs of each participant before the intervention. In addition, we confirmed that they were not sick and sleepless and had no foot injury before initiating the test. In random order, they received two intervention plans: taking a footbath with the *Lu* essential oil (L group) and taking a footbath without any essential oil (control group or C group). All were seated on a chair in the test room during the protocol.

The *Lu* essential oil extracted from the branch of Japanese *Lu* through high-pressure steam distillation was obtained from Kojo Technology (Aomori, Japan). The chemical components of *Lu* essential oil were previously analyzed using gas chromatography-mass spectrometry [17]. The main components are reported to be linalool (42.8%) and 1,8-cineole (13.7%) [17].

### 4.3. Evaluation Index

#### 4.3.1. Questionnaire about the Psychological Assessment

The participants were asked the degrees of comfort, relaxation, and fatigue and the shortened version of the Profile of Mood States (POMS) before and after the intervention. The POMS was developed and validated by McNair et al. in the U.S. It is a self-administered instrument consisting of 65 items designed to identify and assess transient mood states [32]. It measures six mood or affective states: ‘tension–anxiety’, ‘depression–dejection’, ‘anger–hostility’, ‘vigor’, ‘fatigue’, and ‘confusion’. In addition, we calculated the total mood disturbance (TMD) score, which indicates the negative feeling of participants from the POMS score. Yokoyama et al. translated the POMS questionnaire into Japanese and validated it [33]. The shortened version of the POMS questionnaire consists of 30 items and can be completed in only 10 min. The changes in feelings before and after an intervention can be measured. In addition, since the validity of the shortened version is verified [34], we used the shortened version of the POMS questionnaire.

The degrees of comfort, relaxation, and fatigue before and after the footbath were evaluated using a four-point Likert scale: 1, ‘strongly agree’, 2, ‘agree’, 3, ‘disagree’, and 4, ‘strongly disagree’. Additionally, the participants answered an open-ended question regarding impression and feelings toward the footbath with or without the essential oil. In the L group, the participants were asked about the odor intensity, feelings toward using the *Lu* essential oil for the footbath as well as their impressions of using the footbath with the essential oil.

#### 4.3.2. Autonomic Nervous System Activity

During the protocol, the heart rate fluctuations were measured to determine the autonomic nervous system activity using BACS Advance (TAOS Institute, Inc., Kanagawa, Japan) [35,36,37]. The BACS monitor was put on the participants’ right index finger, recorded the heart rate through pulse wave detection, and automatically calculated the high-frequency (HF), low-frequency (LF)/HF ratio, and total power (TP). These values, calculated by heart rate variability analysis, were undertaken based on the fingertip pulse in BACS Advance. The LF value is the power spectrum with a low frequency band of 0.004–0.15 Hz and reflects the activities of both the sympathetic and parasympathetic nervous systems. On the other hand, the HF power spectrum is 0.15–0.4 Hz and reflects only the parasympathetic nervous system activity [38]. Furthermore, the TP is the power of adapting autonomic nervous system and calculated as the sum of the LF and HF power spectra for 5 min.

#### 4.3.3. Aerobic Viable Count

We used a Food Stamp “Nissui” Standard Method Agar Plate (#06050, Nissui Pharmaceutical Co. Ltd., Tokyo, Japan) to estimate the aerobic viable count on the foot. The participants individually stamped their big toe on the agar medium on the plate (10 cm^2^) before and after the footbath with/without the *Lu* essential oil. Thereafter, all samples collected were cultivated in the incubator at 37 °C for 24 h. After incubation, the number of visible colonies was counted.

### 4.4. Protocol

Figure 1 shows the protocol of this study. First, the participants sat on a comfortable chair and rested for 5 min to prevent the influence of any previous action. Then, they filled out the questionnaires, stamped their big toe on the agar medium, and put their right index finger on the BACS monitor for 10 min. Subsequently, they placed their feet in the footbath equipment (PS3871, AIVIL Co., Osaka, Japan) and randomly received a footbath for 5-min with the *Lu* essential oil (i.e., L group) or without the essential oil (i.e., C group). Furthermore, we prepared 4000-mL hot water at 40 °C and used the heat retention function that was attached to the footbath equipment during the procedure. To uniformly dissolve the essential oil in hot water, an 8-mL emulsifier was added. In the L group, 0.5 mL (10 drops) of *Lu* essential oil was used. The C group was treated with hot water containing an emulsifier. After the intervention, the participants sat for 10 min. Finally, they completed the questionnaires and stamped their big toe on the agar medium again. This procedure was repeated a few days later by performing the other kind of footbath with or without *Lu* essential oil.

### 4.5. Statistical Analysis

The POMS subscale scores were computed and compared with the average measurements according to age. Moreover, the data from BACS Advance calculated the mean value of the following three points, 5 min before, during, and after the footbath. A picture of the agar medium cultivated for one day was taken, and the aerobic viable count was visually measured. All data were expressed as the mean ± standard error of the mean and analyzed using the SPSS 16.0 software. Furthermore, the results were compared using the paired t-test, repeated measure ANOVAS and Bonferroni correction, which was multiple comparison. *p* values < 0.05 were considered statistically significant.

## 5. Conclusions

In summary, our study revealed that the footbath with the *Lu* essential oil increased the parasympathetic nervous system activity and released the negative mood states. Furthermore, we suggest that this intervention is a useful nursing care therapy.

## Figures and Tables

**Table 1:** Changes in the POMS scores (n = 20).

POMS Subscale	C Group			L Group		
	Before Intervention	After Intervention	*p* Value	Before Intervention	After Intervention	*p* Value
Tension–anxiety	40.40 ± 4.68	36.25 ± 4.68	0.001 **	42.25 ± 6.99	35.80 ± 2.59	0.000 **
Depression–dejection	41.50 ± 2.93	40.00 ± 2.93	0.017 *	43.05 ± 8.69	40.05 ± 2.35	0.001 **
Anger–hostility	38.60 ± 1.31	38.10 ± 1.31	0.102	40.10 ± 5.04	38.20 ± 0.89	0.010 *
Vigor	42.90 ± 10.90	43.70 ± 10.90	0.959	42.65 ± 10.15	44.55 ± 10.90	0.315
Fatigue	39.30 ± 4.55	35.20 ± 4.55	0.002 **	41.15 ± 7.21	35.40 ± 1.96	0.000 **
Confusion	46.00 ± 6.93	43.25 ± 6.93	0.028 *	47.10 ± 8.48	43.10 ± 4.30	0.000 **
TMD	162.90 ± 15.71	149.10 ± 15.71	0.000 **	171.00 ± 30.45	148.00 ± 12.97	0.000 **

C group: The group of participants who received the footbath without the essential oil, L group: The group of participants who received the footbath with the *Lu* essential oil, POMS: Profile of Mood States. TMD: total mood disturbance, which indicates the negative feelings of the participant from the POMS score. Wilcoxon signed-ranks test; ** *p* < 0.01, * *p* < 0.05.

## Data Availability

The data presented in this study are available in the article.

## Supplementary Materials

Supplementary materials are available online, Figure S1: Structural formula of major components of the *Lindera umbellata* essential oil.
