# Supplementary material for: Psychological and Antibacterial Effects of Footbath Using the Lindera umbellata Essential Oil
Source: Molecules. 2021 Aug 24;26(17):5128. doi: 10.3390/molecules26175128 (PMC8434156; doi:10.3390/molecules26175128)
Supplement: Supplementary file 1 [file molecules-26-05128-s001.zip › molecules-1331972-supplementary.pdf]

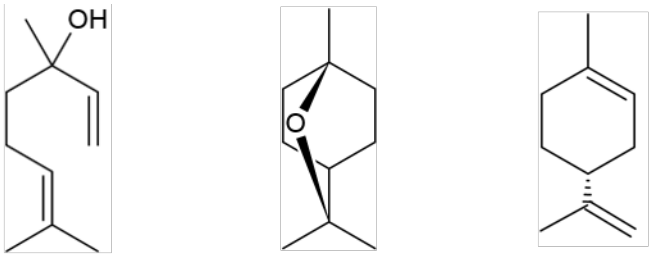

Linalool **1.8-cineole** **D-limonene**

Figure S1. Structural formula of major components of the *Lindera umbellata* essential oil. The main components of the *Lindera umbellata* essential oil used in this study are Linalool (42.8%), 1.8-cineole (13.7%), and D-limonene (7.42%). The structural formula was drawn by ACD/ChemSketch Freeware (ver. 2021.1.0).
